# Alleviating the Mechanical and Thermal Degradations of Highly Sulfonated Poly(Ether Ether Ketone) Blocks via Copolymerization with Hydrophobic Unit for Intermediate Humidity Fuel Cells

**DOI:** 10.3390/polym10121346

**Published:** 2018-12-05

**Authors:** Ae Rhan Kim, Mohanraj Vinothkannan, Chul Jin Park, Dong Jin Yoo

**Affiliations:** 1 Department of Bioenvironmental Chemistry and R&D Center for CANUTECH, Business Incubation Center, Chonbuk National University, Jeollabuk-do 54896, Republic of Korea; 2 Graduate School, Department of Energy Storage/Conversion Engineering, Hydrogen and Fuel Cell Research Center, Chonbuk National University, Jeollabuk-do 54896, Republic of Korea; vinothkannanram@gmail.com (M.V.); top_pcj@hanmail.net (C.J.P.); 3 Department of Life Science, Chonbuk National University, Jeollabuk-do 54896, Republic of Korea

**Keywords:** sulfonated poly(ether ether ketone), hydrophobic oligomer, mechanical stability, intermediate humidity, cell performance

## Abstract

In this contribution, sulfonated poly(ether ether ketone) (SPEEK) is inter-connected using a hydrophobic oligomer via poly-condensation reaction to produce SPEEK analogues as PEMs. Prior sulfonation is performed for SPEEK to avoid random sulfonation of multi-block copolymers that may destroy the mechanical toughness of polymer backbone. A greater local density of ionic moieties exist in SPEEK and good thermomechanical properties of hydrophobic unit offer an unique approach to promote the proton conductivity as well as thermomechanical stability of membrane, as verify from AC impedance and TGA. The morphological behavior and phase variation of membranes are explored using FE-SEM and AFM; the triblock (XYX) membranes exhibits a nano-phase separated morphology. Performance of PEFC integrated with blend and block copolymer membranes is determined at 60 °C under 60% RH. As a result, the triblock (XYX) membrane has a high power density than blend (2X1Y) membrane.

## 1. Introduction

Polymer electrolyte fuel cells (PEFCs) have efficacy as next-generation energy devices owing to their features of extraordinary energy density from single-step energy conversion and environmental benignity due to low pollutant-emission levels [1,2,3,4]. However, PEFCs have faced a high-cost key material dilemma, which is the main factor hindering their widespread utilization. The proton exchange membrane (PEM), the key module of PEFCs, acts as a proton conducting medium [5,6,7,8]. At present, Nafion (Dupont) is the most commonly used PEM in industry as it shows high proton conductivity, structural stability, unique micro-morphological structure, and mechanical toughness [9]. The key drawbacks of this membrane, however, are limited proton conductivity while operating under anhydrous environment, synthesis difficulty, environmental unfriendliness, and high cost [10,11,12]. The two most common methods used to solve these issues are (i) modification of Nafion using other polymers or inorganic compounds and (ii) designing new PEMs that are free of Nafion [13,14,15,16]. The former strategy typically suffers due to incompatibility between different components. The second approach, which is more favorable for fabricating commercial PEMs at an industrial scale involves investigating non-fluorinated polymers include sulfonated poly(ether ether ketone) (SPEEK) [17], sulfonated poly(imide) [18], sulfonated poly(arylene ether ketone) [19], and sulfonated poly(phenylene sulfone) [20]. Unfortunately, aromatic polymer-based PEMs face an important struggle, i.e., imbalance between membrane conductivity and mechanical toughness [21]. For instance, SPEEK is the most favorable substitute to Nafion owed to its thermal stability, mechanical integrity, modification flexibility, and low cost [22]. Nevertheless, the ionic channels of SPEEK are narrower, disordered, and unconnected; thus, the maximized proton conductivity can only be achieved via increasing the degree of sulfonation (DS) [23]. PEMs with a high DS exhibit poor physical integrity and high water swelling behavior, resulting in rapid degradation of PEFC performance [24]. On other hand, chemical stability is an important factor that is highly confined to SPEEK in general. In OH and OOH radical environments, the durability of SPEEK membranes falls to a critically low level due to chemical unzipping of polymer chains. To address the aforementioned issues, various strategies such as introducing cross-linkers, blending hydrophobic polymers, or physically reinforcing with metal-ion scavengers have been proposed to improve durability [9,25,26]. Since mechanical brittleness and proton conductivity decline when applying the aforementioned strategies, block copolymerization of SPEEK could retain its full advantages as well as further boost the performance of PEMs. In our previous report, the physical blending of SPEEK with block copolymer exhibited a 10% increase in ion conductivity [27]. However, physical blending is based on non-covalent interactions that are difficult to ideally arrange two polymer matrixes and construct conducting channels in PEMs. Therefore, a desirable strategy needs to be explored to further increase the ion conductivity of PEMs. Block copolymerization of SPEEK, derived from commercially available PEEK, with a hydrophobic unit is a promising strategy to alleviate the drawbacks existing in the physical blending of PEMs.

Herein, in consideration of the practicability of PEMs, we explored a nucleophilic substitution strategy to synthesize a block structure wherein SPEEK (X) was the hydrophilic unit and fluorinated poly (arylene propane biphenyl) (FPAPB) (Y) was the hydrophobic unit. Further, a series of block structures (XY, XYX, and YXY) were synthesized to determine the best combinations and ratios. These polymers can simply accessible via reported synthetic routes. The acquired block copolymers were intensively characterized as mentioned in literature. Based on these findings, we show that even chemically inter-connecting SPEEK using a hydrophobic unit can be a better approach; thus, the corresponding PEM should be a potential candidate for integration into PEFC devices. Additionally, effects on chemical structure (^1^H-NMR), phase-separation morphology (FE-SEM), and physiochemical properties including thermal and mechanical stability (TGA, DSC, and UTM) and proton conductivity (AC impedance test), were also investigated. To further examine the effect on PEFC performance, an MEA was fabricated, and a polarization curve was investigated.

## 2. Materials and Methods

### 2.1. Materials

Decafluorobiphenyl (DFBP, 98%) and 4,4′-hexafluoroisopropylidenediphenol (HFIP, 99%) were acquired from Sigma-Aldrich. Poly(ether ether ketone) (PEEK) pellet was purchased from Victrex. Dimethyl sulfoxide (DMSO, 99.8%), *N*,*N*-dimethylacetamide (DMAc, 99.8%), potassium carbonate (K_2_CO_3_, 99.5%), toluene (99.8%), *N*-methylpyrrolidinone (NMP, 99%), and tetrahydrofuran (THF, 99%) were procured from Sigma-Aldrich.

### 2.2. Synthesis of Na Salt Form of X

Synthesis of the Na salt form of X was performed as reported in the literature [28]. Commercial PEEK (5 g) and sulfuric acid (95 mL) were added to a 200 mL two-necked flask equipped with a mechanical stirrer and heated on a hot plate at 50 °C. By changing the reaction time, SPEEK with varied degrees of sulfonation was acquired. After the reaction, the resulting solution was decanted into deionized (DI) ice water. The settled-down fibrous product was washed with DI water up to the filtrate reached pH 7. The dry X was submerged in 0.1 M NaCl solution for 24 h to interchange H^+^ ions by Na^+^ ions. The Na salt form of X was dried again and kept for further usage.

### 2.3. Synthesis of Y 

HFIP (2.017 g, 6 × 10^–3^ M), DFBP (2.0 g, 6 × 10^–3^ M), K_2_CO_3_ (1.66 g, 12 × 10^–3^ M), DMAc (11 mL), and toluene (20 mL) were placed in a clean 100 mL three-necked round bottomed (RB) flask equipped with a condenser with N_2_ gas valve, and a magnetic stir bar. The polymerization reaction was carried out at 120–140 °C (oil bath) under N2 with constant stirring for 3 h. To remove produced water, the temperature of the reaction was increased to 160 °C. The reaction was continued for 24 h to acquire a light yellow viscous liquid mixture, which was cooled and precipitated in a mixture of DI water, methanol, and acetone. The obtained fibrous precipitate was successively washed with DI water few times and kept in a vacuum oven at 70 °C for 12 h.

### 2.4. Synthesis of Diblock and Triblock Copolymers

For the preparation of XY, Na salt form of X (0.63 g, 4.78 × 10^–6^ M, *M_W_* = 132,000), Y (0.134 g, 4.78 × 10^–6^ M, *M_W_* = 28,000), potassium carbonate (0.026 g, 1.90 × 10^–4^ M, *M_W_* = 138), DMAc (9 mL), and toluene (19 mL) were placed in a three-necked RB flask. The reaction involves two-step process (i) dehydration of system in which the mixture was heated with reflux at 120–140 °C for 10 h and (ii) polymerization of oligomers in which the reaction temperature was retained at 160 °C for 14 h. Finally, the reaction mixture was purified by repeated washing with co-solvent (methanol/acetone/deionized water, 10:1:1, v/v/v) and separated by filtration. To ensure the complete elimination of unreacted hydrophobic oligomers and formation of block copolymer, the product was further washed with dichloromethane. The XYX and YXY polymers were fabricated using a similar strategy by varying the ratio of precursors. For the XYX case, X (1.26 g, 9.56 × 10^–6^ M, *M_W_* = 132,000), Y (0.134 g, 4.78 × 10^–6^ M, *M_W_* = 28,000), potassium carbonate (0.0524 g, 3.80 × 10^–4^ M, *M_W_* = 138), DMAc (11 mL), and toluene (19 mL) were used. For the YXY case, X (0.63 g, 4.78 × 10^–6^ M, *M_W_* = 132,000) and Y (0.269 g, 9.56 × 10^–6^ M, *M_W_* = 28,000), potassium carbonate (0.0524 g, 3.80 × 10^–4^ M, *M_W_* = 138), DMAc (11 mL), and toluene (19 mL) were used.

### 2.5. Membrane Preparation

**Figure 1:**
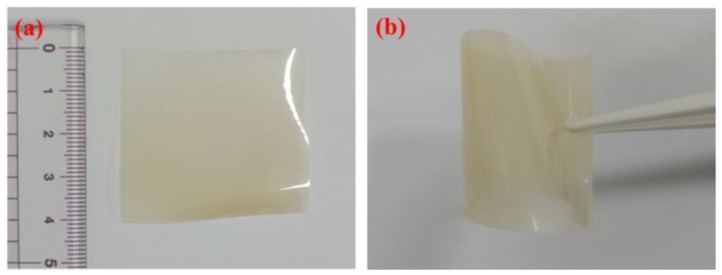
Digital photographs XYX membrane (**a**) flat view and (**b**) folded view.

A preferred amount of X, XY, XYX, or YXY was added to DMF, and the resulting suspension was sonicated to acquire a homogeneous solution. After further stirring for 72 h at 60 °C, the mixture was cast onto a glass plate and kept in a 120 °C oven for 6 h for solvent evaporation. Similarly, the blend membrane was prepared by blending X and Y in DMF with the ratio of 1:1, 2:1, or 1:2. The acquired membranes were changed to acidic form by submerging in 1 M H_2_SO_4_ and then rinsed in DI water to detach free acids. Figure 1 shows the digital photographs of the XYX membrane wherein the fabricated XYX membrane is flexible without any significant visible defects.

## 3. Characterizations

The as-made membranes were analyzed with field-emission scanning electron microscopy (FE-SEM) (SUPRA 40VP, ZEISS, city, state, country), atomic force microscopy (AFM) (multimode-8 model, Bruker, city, state, country), Fourier transform nuclear magnetic resonance spectroscopy (FT-NMR), small angle X-ray scattering (SAXS) (EMPYREAN, Malvern Panalytical, city, state, country), thermogravimetric analyzer (TGA) (TA-instruments-Q20), differential scanning calorimetry (DSC) (TA-instruments-Q50), and universal tensile machine (UTM) (model 5565, Lloyd, Fareham, UK).

## 4. Measurements

### 4.1. Water Uptake

Water retention properties of membranes were measured at 30 °C. The dry membranes were pre-weighed and submerged in DI water for 24 h at 30 °C. Next, the membranes were removed, wiped with tissue paper, and weighed again [29]. The water uptake values were calculated from mass difference of dry and wet membranes with the following equation

(1) $$\mathrm{Water}\;\mathrm{uptake}\;\left(\%\right)=\left[\frac{M_{wet}-M_{dry}}{M_{dry}}\right]100$$

The wet membrane mass (*M_wet_* in grams) and dry membrane mass (*M_dry_* in grams) were acquired using a Denver four digit counter balance (Model: S-234) with a precision of ±0.01 mg.

### 4.2. Dimensional Stability

Dimensional stability of membranes was determined from the volume change before and after uptake of water. The following equation was used to calculate the swelling ratio

(2) $$\mathrm{Swelling}\;\mathrm{ratio}\;\left(\%\right)=\left[\frac{V_{wet}-V_{dry}}{V_{dry}}\right]100$$

where *V_dry_* and *V_wet_* are the volumes of the membrane samples before and after water uptake, respectively.

### 4.3. Contact Angle

To determine the wettability of membranes, contact angle measurements were performed using a drop shape analyzer (DSA10, Kruss GmbH, city, Germany).

### 4.4. Proton Conductivity 

Temperature dependent conductivity of hydrated samples was scrutinized using alternating current impedance spectroscopy (Sci Tech instrument equipped with Keithley-2400 source meter). The measurement was performed in a Bekk-Tech cell in which the membrane was placed across four Pt electrodes [30]. The conductivity was calculated from the equation given below

(3) $$\sigma \;\left(mS\;cm^{-1}\right)=\left[\frac{L}{RTW}\right]$$

where the distance between Pt probes is *L* (cm), while ionic resistance of the cell is *R* (Ω), and the width and thickness of the membrane sample are designated as *W* (cm) and *T* (cm), respectively. Membrane reproducibility toward conductivity was verified by performing the measurement twice.

## 5. Membrane Electrode Assembly (MEA) Preparation and PEFC Testing

Carbon papers attached with diffusion layers were employed as electrodes. For preparation of the anode layer or cathode layer, an appropriate amount of Pt/C catalyst (40%, Johnson Matthey Co.) was dispersed in ionomer solution (isopropyl alcohol with Nafion ionomer) and applied onto the carbon paper surface using spray gun. A loading Pt catalyst was controlled to be 0.3 mg cm^–2^ for both electrodes. MEAs with an active surface area of 5 cm^2^ were acquired via sandwiching the membrane between anode and cathode and subsequently pressed 14,000 psi at 90 °C for 3 min. Each acquired MEA was then fitted with gaskets and integrated in a PEFC cell fixture. Afterwards, PEFC cell fixture was connected with Horizon (Sci Tech, South Korea) test station and performance curves were extracted as a function of current density. The H_2_/O_2_ PEFC was operated at a flow rate of 100 ccm min^−1^ for H_2_ and 400 ccm min^−1^ for O_2_ at 60 °C and 60% RH.

## 6. Results and Discussion

### 6.1. Synthesis and Structural Properties

**Figure 2:**
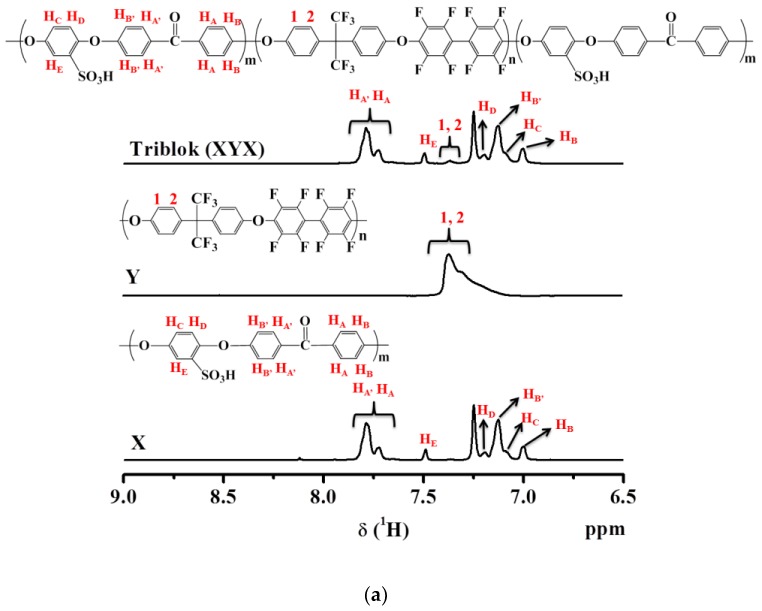

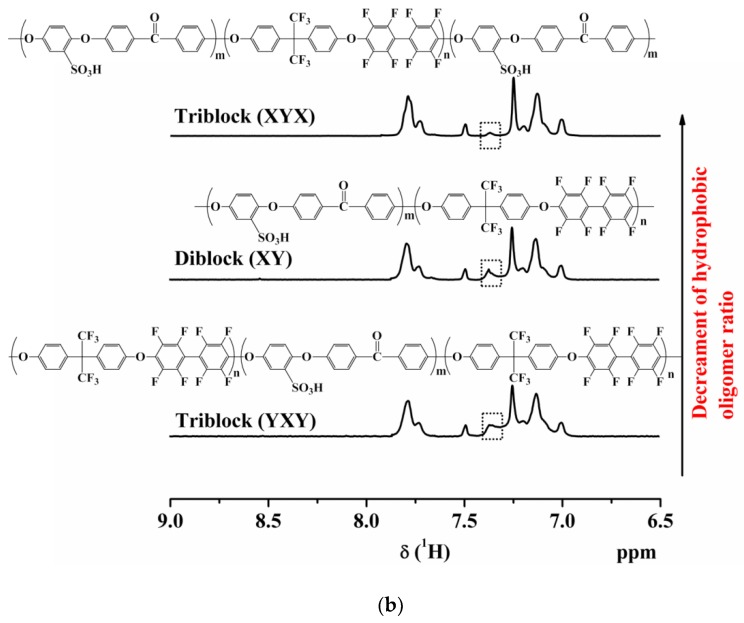
(**a**) ^1^H-NMR spectra of X, Y and XYX. (**b**) ^1^H-NMR spectra of block copolymers.

Block copolymers were produced by polycondensation and polymerization methods as described previously. As the concentration of SO_3_H groups is directly related to conductivity, to study the correlation, the content of the sulfonated block with a range of lengths was also considered. SPEEK and hydrophobic oligomer were utilized as precursors for synthesis of multi-block copolymers (XY, XYX, and YXY). To determine the chemical structures, the as-made X, Y, triblock copolymers (XYX and YXY), and diblock copolymer (XY) were dissolved in DMSO-d_6_ solvent and analyzed by ^1^H-NMR spectroscopy (Figure 2a,b). X peaks are observed from 6.9 to 7.9 ppm in the ^1^H-NMR spectrum, which are associated with aromatic protons. While the successive grafting of SO_3_H groups in X could be endorsed from the peak at 7.5 ppm. By evaluating the ratio of peak area at 7.5 ppm to the total peak area of remaining peaks, the DS of SPEEK was determined. Triblock copolymers (XYX) had significant signals in the range of 6.9 to 7.9 ppm, which arose from the aromatic protons of SPEEK and the hydrophobic units. In other hand, the decrement of signal intensity at 7.3 ppm in the order of YXY, XY, and XYX indicates the decrement in hydrophobic ratio in the polymer. It is noteworthy that the XYX polymer had a very small quantity of hydrophobic component (Figure 2b). As shown in Table 1, the SPEEK (X) and hydrophobic polymer (Y) had considerable molecular weights (*M_w_*) of 131,700 and 28,000, respectively. The ratio of X and Y in the triblock polymer was controlled to be 2:1 or 1:2, and the diblock polymer was controlled to be 1:1 molar ratio. The average *M_w_* values of XYX, XY, and YXY were 183,400, 155,700, and 179,700 respectively. The PDI (*M_w_*/*M_n_*) values for XYX, XY, and YXY were 4.0, 3.6, and 3.5, respectively. In addition, the morphology of the as-made membranes was verified by SAXS, and the ionic cluster dimensions are compared in Figure S1. The SAXS patterns revealed similar morphologies for the XYX, XY, and YXY membranes, which differed from the blend membrane.

### 6.2. Morphological Behaviors

**Figure 3:**
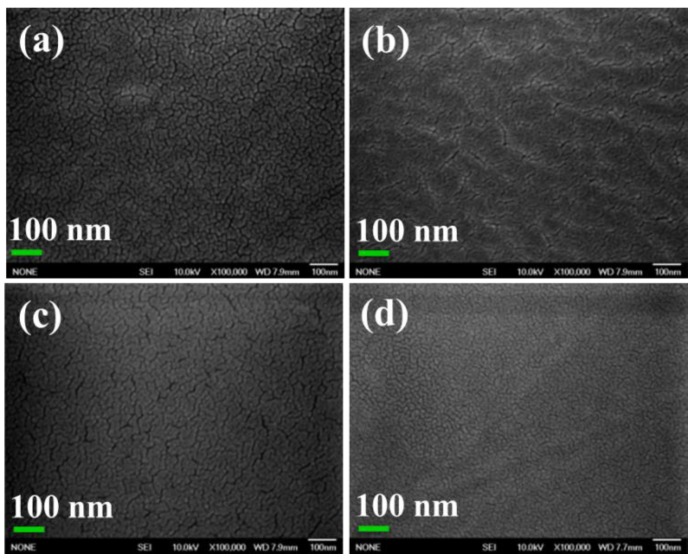
FE-SEM images of membranes of (**a**) X;(**b**) XY; (**c**) XYX; and (**d**) YXY.

**Figure 4:**
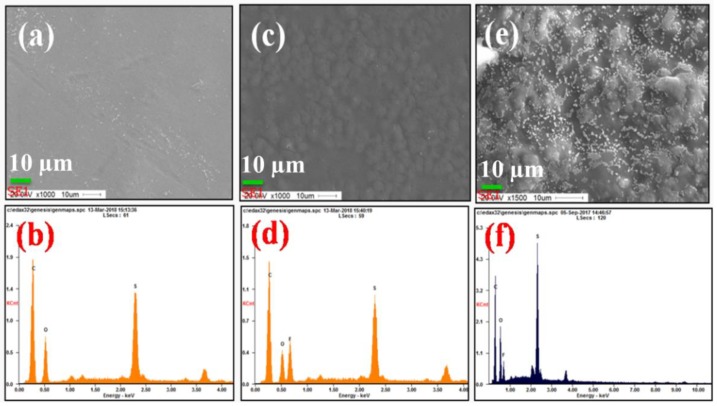
FE-SEM images and EDX spectra of membranes of (**a**,**b**) X; (**c**,**d**) triblock (XYX); (**e**,**f**) blend (2X1Y).

**Figure 5:**
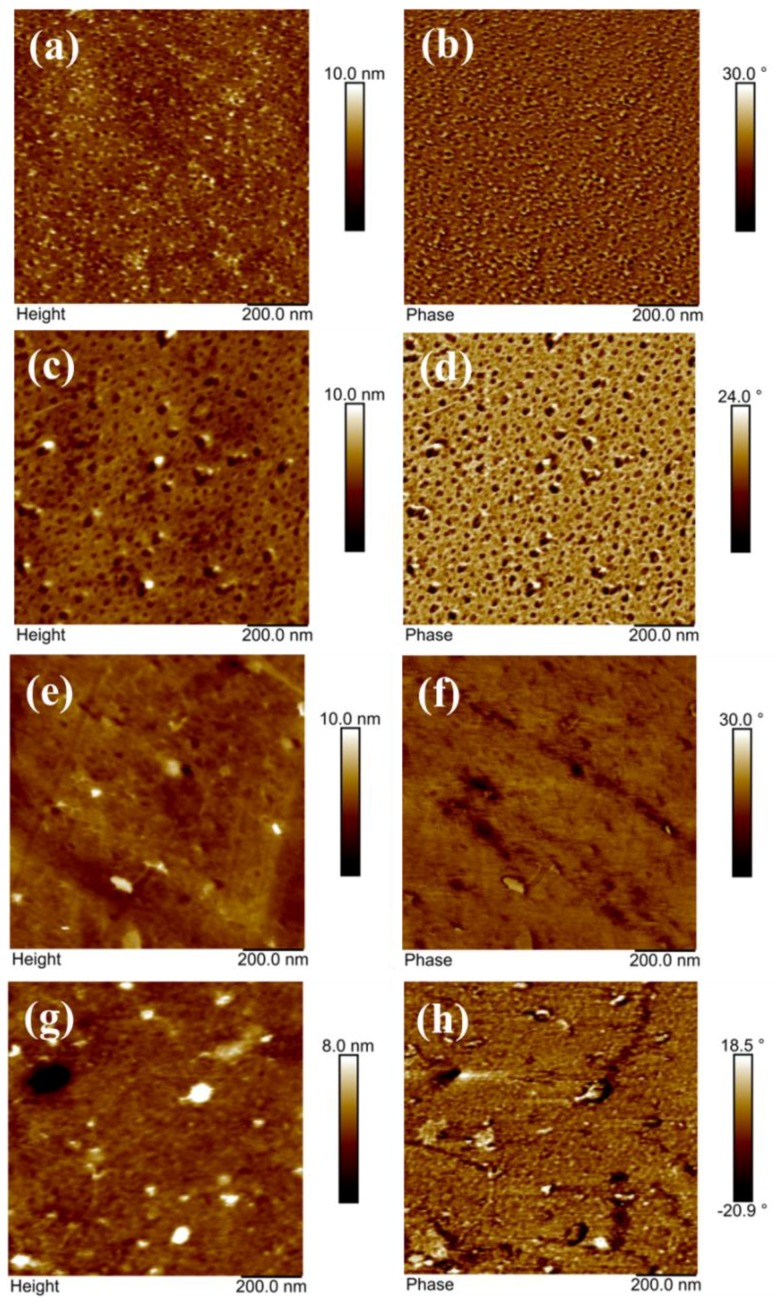
AFM height and phase images of membranes of (**a**,**b**) X; (**c**,**d**) XY; (**e**,**f**) XXY; and (**g**,**h**) YXY.

**Figure 6:**
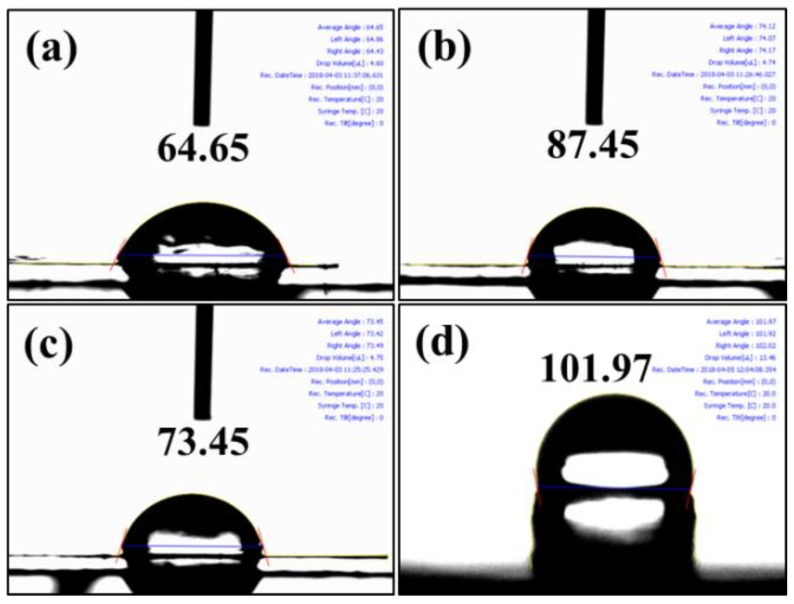
Water contact angle images of (**a**) X; (**b**) XY; (**c**) XYX; and (**d**) YXY.

FE-SEM morphologies of as-made XYX, XY, and YXY type membranes are given in Figure 3, each of which has a rough surface and uniform hydrophilic-hydrophobic phase-separation. In X membrane (Figure 3a), two types of patterns can be clearly observed: (i) hydrophilic cluster and (ii) hydrophobic cluster. When compared to X, phase contrast in the XY membrane is found to be increased due to two dissimilar material domains (Figure 3b). The higher SO_3_H density in the X part produced brighter domains, while the darker regions indicate the hydrophobic component [31]. The degree of phase-separation varies for XY, XYX, and YXY due to the various ratios of hydrophilic and hydrophobic components. As shown in Figure 4, the blend (2X1Y) shows the aggregated particles due to the immiscibility of hydrophilic and hydrophobic units (Figure 4e), whereas the triblock (XYX) membrane exhibits the homogeneous distribution caused by the covalent bond formation to make the single polymer unit (Figure 4c). In AFM (Figure 5), a higher quantity of phase-separation was detected for the XY type membrane compared to XYX and YXY type membranes. This is because of the equal ratio of hydrophilic and hydrophobic oligomers. The water contact angle of the membranes decreased according to increasing the X ratio in the block structure (Figure 6).

### 6.3. Thermal Stabilities

**Figure 7:**
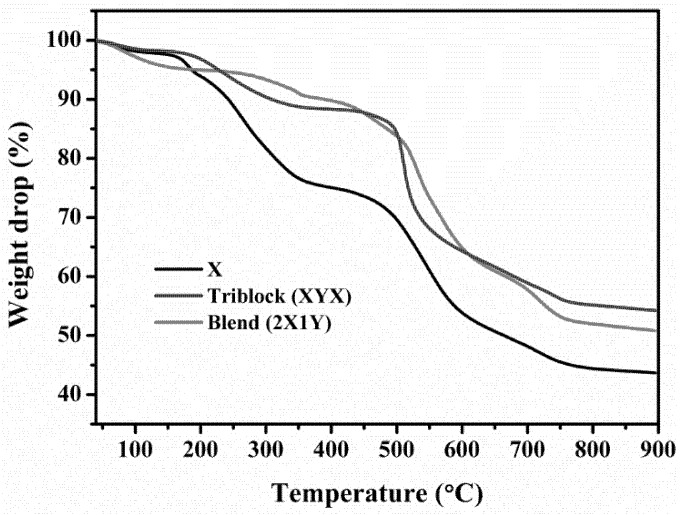
TGA curves of X, blend and block copolymer membranes.

Figure 7 exhibits TGA curves for determining the percent weight loss. At a temperature of 800 °C, the SPEEK membrane had a residual weight of 43.6%, which may be due to rapid decomposition of the main skeleton, while the XYX membrane had a residual weight of 45.8%, the XY membrane had a residual weight up to 51.9%, and the YXY membrane had a residual weight of 53.07% at the same temperature. These results reveal that the XYX and YXY membranes are thermally less stable compared to the YXY membrane. DSC curves of the as-made membranes showed considerable endothermic inflection (T_g_) values, all between 192–218 °C (Figure S2). The XYX type had a higher T_g_ compared to the other membranes. In general, the T_g_ of a membrane increases with the number of ionic sites on the polymer chains because of an ionomer effect. The T_g_ of the XYX membrane was 218.15 °C, which is greater by a factor of 1.01 and 1.07 compared to the XY (216.13 °C) and YXY (204.15 °C), respectively. Meanwhile the great density of SO_3_H sites increases the molecular bulkiness of the polymers, the T_g_ of membranes increases according to the bulkiness of the membrane. A high density of SO_3_H groups generates large numbers of internal hydrogen bonds that restrict reorganization of polymer chains during heating, resulting in a decreased T_g_ for the XY and YXY membranes.

### 6.4. Mechanical Properties

**Figure 8:**
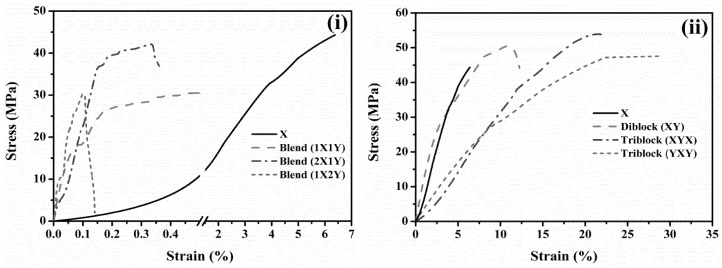
UTM stress-strain curves of (**i**) blend and (**ii**) block copolymer membranes.

Generally, the interactions between polymer backbones can be weaker due to absorbed free water molecules. Accordingly, the tensile stiffness of PEMs is lower in the wet condition compared to dry condition. Therefore, the tensile stiffness and elongation break of the membranes were measured in dry condition at room temperature (Figure 8). The mechanical integrity of PEEK is admirable in dry state; however, once the PEEK chain was grafted with SO_3_H groups, a lower mechanical integrity was occurred due to the plasticization effect. Tensile strength and elongation break for SPEEK membrane in are 44.5 MPa and 6.5%, respectively. Both of them were enhanced significantly after attachment of the hydrophobic oligomer via block copolymerization. These results demonstrate that the prepared diblock (XY) and triblocks (XYX and YXY) are sufficiently robust for fuel cell applications. Nevertheless, corresponding blend membranes show lower tensile strength and elongation break.

### 6.5. Proton Conductivity, Arrhenius Plot, and Water Uptake

**Figure 9:**
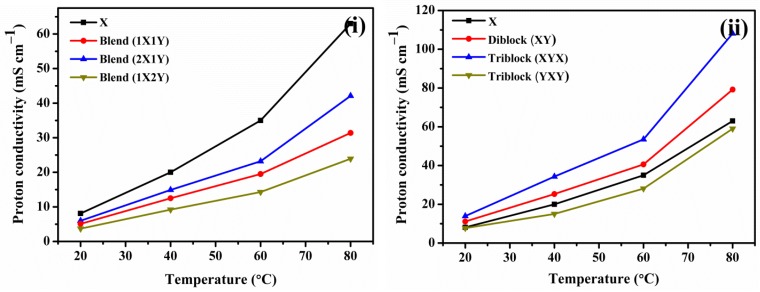
Proton conductivity plots of (**i**) blend and (**ii**) block copolymer membranes measured under 100% RH.

The proton conductivities of block copolymer and blend membranes were measured to investigate the hydrophobic oligomer effects (Figure 9). Conductivity of the triblock (XYX) membrane, 13.9 mS cm^−1^ to 108.1 mS cm^−1^ when increase the temperature from 20 to 80 °C, was significantly greater than that of the diblock (XY) membrane, 11.1 mS cm^−1^ to 79.2 mS cm^−1^. The triblock (YXY) type displayed a maximum conductivity of 59.2 mS cm^−1^ at 80 °C due to the existence of a low hydrophilic ratio in PEM compared to the XYX and XY membranes. On other side, the conductivity of block copolymer membranes is better compared to blend membranes, which might be due to the regular architecture of conducting channels caused by multi-block structure. Since the blend membranes have more –SO_3_H groups, it seems the conducting channels of blend membranes were not regular and wide. For the case of X, the conducting channels are narrower and unconnected. Thus, the conductivity of X is lower even when the degree of sulfonation is higher. The conductivity of XYX and XY membranes is also better compared to X membrane, which is due to the ordered and extended conducting channels caused by block structure. Activation energies (E_a_) of the ion conductivity values were calculated from Arrhenius plots using the Arrhenius formula [32,33,34,35,36].

$$\ln\sigma =\ln\sigma_{0}-\frac{E_{a}}{RT}$$

where *σ* and $\sigma_{0}$ are proton conductivity and pre-exponential factor, respectively (in mS/cm), *E_a_* is the activation energy (in kJ/mol), *R* is the gas constant (in J/molK), and *T* is the absolute temperature (in K). The XYX, XY, and YXY demonstrated E_a_ of 15.1, 18.3 and 20.5 KJ mol^−1^, respectively (Table 2). The water retention properties of the membranes were evaluated from the weight change of the membrane before and after water uptake (Table 2). YXY is a more hydrophobic type of copolymer membrane, which can absorb only a small volume of water, while a larger number of ionic sites are responsible for higher water intake of the XYX membrane [37]. The swelling ratio of membranes is directly proportional to water uptakes. XYX membrane exhibits high swelling due to high water uptake (Table 2).

### 6.6. PEFC Performance

**Figure 10:**
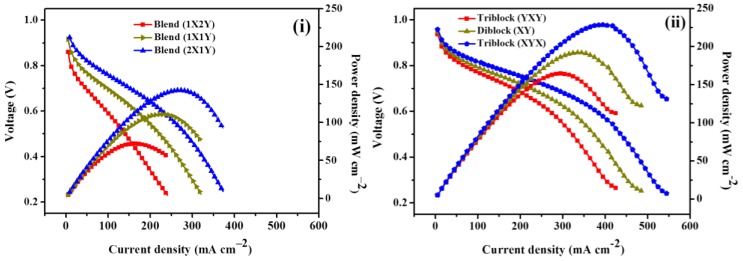
H_2_/O_2_ PEFC curves of (**i**) blend and (**ii**) block copolymer membranes evaluated at 60 °C under 60% RH.

Figure 10 presents the single cell activities of PEFC comprised of blend and block copolymer membranes at 60 °C under intermediate (60%) RH and atmospheric pressure. The good open circuit voltage of block copolymer (YXY, XY, and XYX) membranes indicates that H_2_ permeability across the membrane is lesser, which might the avoid loss of fuel and mixed potentials with O_2_. The triblock (XYX) membrane delivered a peak power density of 229 mW cm^–2^ at a load current of 393 mA cm^–2^, which is 1.6 times greater than that of the blend (XYX) membrane that delivered only a peak power density of 142 mW cm^–2^ at a load current of 287 mA cm^–2^. The excess of ion conducting channels in the multi-block copolymer structure performs as a proton conducting medium, which enables the membrane to achieve high proton conductivity. The intermolecular hydrogen bonds formed between SO_3_H groups of the triblock (XYX) chains assist to form complex structures along the cross section of the membrane, blocking the passage of H_2_ gas and reducing H_2_ gas permeability. A cumulative effect of the aforesaid two factors is responsible for the high power density of the triblock (XYX) membrane. Table 3 demonstrates the comparison table of current study with other literatures. 

## 7. Conclusions

In summary, we present novel copolymer structures containing SPEEK (derived from commercially available PEEK) and artificial hydrophobic units to boost the thermal and mechanical properties of PEMs. Compared to blend (XYX), such a novel polymer structure of optimized triblock (XYX) membrane has brought a significant improvement in thermal stability. Being extended and continuously connected SPEEK chains formed in the XYX PEMs, the mechanical strength was elevated obviously. The triblock (XYX) constructs successive and effective conducting channels, which dramatically increase the proton conductivity of the membrane. In particular, the triblock (XYX) structure exhibits extended ion transport channels and shorter ion transport distances compared to traditional SPEEK and blend (2X1Y) PEMs, thus the PEFC performance of triblock (XYX) membrane was elevated significantly even under 60% RH. This work presents informative data relevant to the synthesis of new block copolymer structures containing SPEEK for use in high performance PEMs.

## Figures and Tables

**Table 1:** GPC analysis of prepared polymers

Polymer	Area (%)	*M_n_*	*M_w_*	*M_z_*	*M_w_*/*M_n_* (PDI)
X	92.7	50,600	131,700	307,200	2.6
Y	70.5	6600	28,000	66,300	4.2
XYX	94.8	45,700	183,400	605,000	4.0
XY	95.8	42,900	155,700	458,200	3.6
YXY	96.5	50,400	179,700	546,400	3.5

**Table 2:** Water uptake, swelling ratio, and activation energy of prepared membranes

Membrane	Water Uptake (%)	Swelling Ratio (%)	Activation Energy (E_a_)
X	23.1	17.6	11.3
XYX	18.3	13.6	15.1
XY	12.2	9.4	18.3
YXY	7.6	4.4	20.5

**Table 3:** Comparison table of performance of various block copolymer PEMs

Membrane	Peak Current Density (mA cm^–2^)	Peak Power Density (mW cm^–2^)	Operating Conditions		Reference
			Relative Humidity (%)	Temperature (°C)	
Triblock (XYX)	550	229	60	60	This work
Block-30	-	-	-	-	[30]
sPAS-12/4	980	-	100	70	[38]
SPB/PAE	1200	-	100	80	[39]
sPP-b-PAES (6.5k)-3.0	1920	780	100	70	[40]
sPEEK-b-sPB (I-4)	-	-	-	-	[41]
SMBP-17	1210	410	100	80	[42]

## Supplementary Materials

The following are available online at http://www.mdpi.com/2073-4360/10/12/1346/s1, Figure S1: SAXS patterns of SPEEK, blend and block copolymer membranes; Figure S2: DSC curves of SPEEK and block copolymer membranes.
