# Supplementary material for: Alleviating the Mechanical and Thermal Degradations of Highly Sulfonated Poly(Ether Ether Ketone) Blocks via Copolymerization with Hydrophobic Unit for Intermediate Humidity Fuel Cells
Source: Polymers (Basel). 2018 Dec 5;10(12):1346. doi: 10.3390/polym10121346 (PMC6401815; doi:10.3390/polym10121346)
Supplement: Supplementary file 1 [file polymers-10-01346-s001.pdf]

Supplementary Materials: Alleviating the Mechanical and Thermal Degradations of Highly Sulfonated Poly(Ether Ether Ketone) Blocks via Copolymerization with Hydrophobic Unit for Intermediate Humidity Fuel Cells

Ae Rhan Kim, Mohanraj Vinothkannan, Chul Jin Park and Dong Jin Yoo

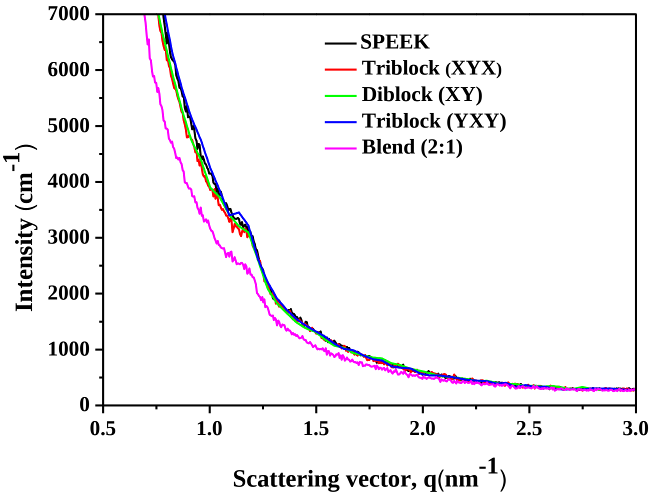

Figure S1. SAXS patterns of SPEEK, blend and block copolymer membranes.

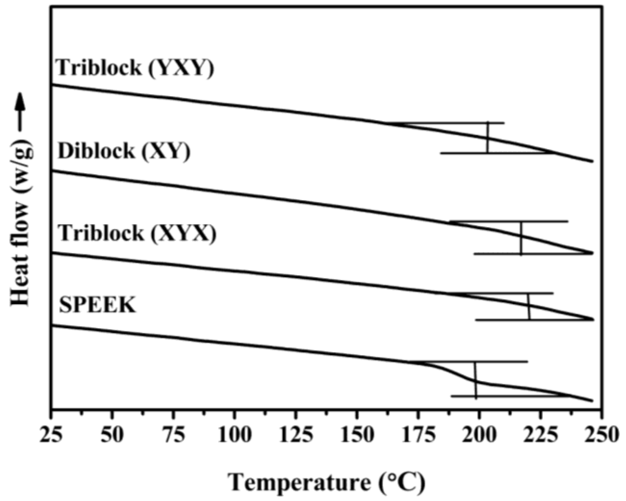

Figure S2. DSC curves of SPEEK and block copolymer membranes.
